# [1,1-(Butane-1,4-diyl)-2,3-dicyclohexylguanidinato]dimethylaluminum(III)

**DOI:** 10.1107/S1600536810046787

**Published:** 2010-11-20

**Authors:** Haoyang Li, Yonggang Xiang, Hongfei Han

**Affiliations:** a Department of Chemistry, Taiyuan Normal University, Taiyuan 030031, People’s Republic of China

## Abstract

In the crystal structure of the title complex, [Al(CH_3_)_2_(C_17_H_30_N_3_)], the Al^III^ cation is coordinated by two methyl ligands and two N atoms from the guanidinato ligand in a distorted tetra­hedral geometry. The dihedral angle between the CN_2_ and AlC_2_ planes is 85.37 (2)°. The two N atoms of the guanidinato ligand exhibit an almost uniform affinity to the metal atom.

## Related literature

For related guanidinato compounds, see: Chandra *et al.* (1970 ▶); Coles & Hitchcock (2004 ▶); Corey *et al.* (2006 ▶); Zhou *et al.* (2007 ▶). For related *ortho* metalation reactions, see: Kondo *et al.* (2007 ▶).
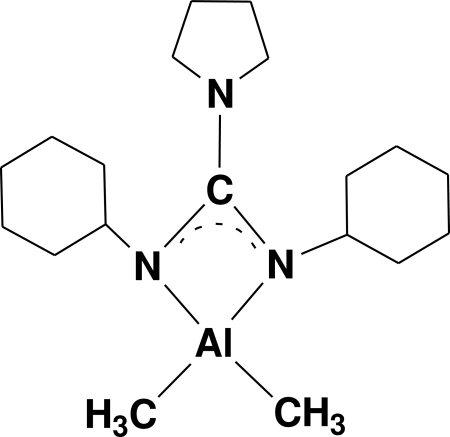


## Experimental

### 

#### Crystal data

[Al(CH_3_)_2_(C_17_H_30_N_3_)]
*M* *_r_* = 333.49
Orthorhombic, 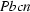

*a* = 18.263 (4) Å
*b* = 10.596 (2) Å
*c* = 10.449 (2) Å
*V* = 2022.0 (7) Å^3^
*Z* = 4
Mo *K*α radiation
μ = 0.11 mm^−1^
*T* = 293 K
0.40 × 0.30 × 0.30 mm

#### Data collection

Bruker SMART CCD area-detector diffractometer
Absorption correction: multi-scan (*SADABS*; Sheldrick, 1996 ▶) *T*_min_ = 0.959, *T*_max_ = 0.969
7156 measured reflections
1772 independent reflections
1630 reflections with *I* > 2σ(*I*)
*R*_int_ = 0.059

#### Refinement

*R*[*F* ^2^ > 2σ(*F* ^2^)] = 0.095
*wR*(*F* ^2^) = 0.232
*S* = 1.42
1772 reflections
107 parameters
H-atom parameters constrained
Δρ_max_ = 0.31 e Å^−3^
Δρ_min_ = −0.43 e Å^−3^

### 

Data collection: *SMART* (Bruker, 2000 ▶); cell refinement: *SAINT* (Bruker, 2000 ▶); data reduction: *SAINT*; program(s) used to solve structure: *SHELXS97* (Sheldrick, 2008 ▶); program(s) used to refine structure: *SHELXL97* (Sheldrick, 2008 ▶); molecular graphics: *DIAMOND* (Brandenburg, 1999 ▶); software used to prepare material for publication: *SHELXL97*.

## Supplementary Material

Crystal structure: contains datablocks I, global. DOI: 10.1107/S1600536810046787/jh2223sup1.cif

Structure factors: contains datablocks I. DOI: 10.1107/S1600536810046787/jh2223Isup2.hkl

Additional supplementary materials:  crystallographic information; 3D view; checkCIF report

## Figures and Tables

**Table:** 

Al—N1	1.922 (4)
Al—C18	1.961 (6)

**Table:** 

N1—Al—N1^i^	69.8 (2)
C18^i^—Al—C18	114.2 (4)

Symmetry code: (i) 
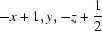
.

**Fig. 1.**
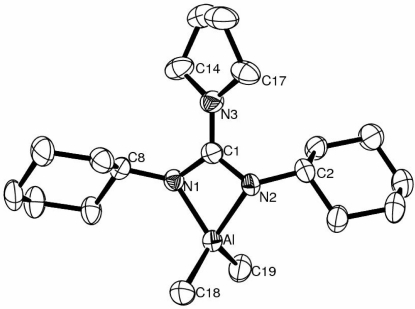
The molecular structure, showing the atom–numbering scheme. Displacement ellipsoids are drawn at the 30% probability level. H atoms are presented as a small spheres of arbitrary radius.

## supplementary crystallographic information

### Comment

Since the first guanidinato complexes have been reported in 1970 by Lappert *et al.* (Chandra *et al.*, 1970), guanidinato ligands have been used extensively in the coordination chemistry of transition, f-block, and main-group metals (Corey *et al.*, 2006). Moreover many guanidinato complexes were reported showing good performance in ethylene polymerization (Zhou *et al.*, 2007) and in Ring-Opening Polymerisation (Coles & Hitchcock, 2004). It implied that the guanidinato complex would behave better in catalysis application.

There has been a great deal of research in directed ortho metalation reactions (Kondo *et al.*, 2007). We had expected guanidinato lithium, the result of the addition of N,N'-dicyclohexyl carbodiimide with N-tetrahydropyrrolyl lithium, when reacting with trimethyl aluminum, to produced a new kind of complex containing Al and Li atoms. However, X-ray diffraction on the complex obtained in the reaction revealed that the Li atom was replaced by Al atom surprisingly. Its molecular structure is shown in Fig. 1. In the molecular structure of the complex, the metal atom is chelated with the guanidinato ligand. The four-coordinate Al(III) center demonstrates a highly distorted tetrahedral geometry. The distances from the two N atoms to Al atom are almost equal [N1-Al: 1.918 (4) Å, N2-Al: 1.925 (4) Å]. It indicates that the two N atoms of guanidinato ligand exhibit almost uniform affinity to the metal center.

### Experimental

A solution of N-tetrahydropyrrolyl lithium in diethylether (0.232g, 3mmol) was added dropwise with stirring at 273K to a solution of 0.619g (3mmol) of *N, N*'-dicyclohexyl carbodiimide in ether. The mixture was warmed to room temperature and stirred for 2h. A 2M solution of trimethylaluminum in heaxene (1.5mL, 3mmol) was added at 195K to the mixed solution. The mixture was warmed to room temperature and stirred for 12h. Concentration of the filtrate under reduced pressure produced the colorless crystals suitable for X-ray analysis 3 days later (yield 0.620g, 62%).

### Refinement

Positional parameters of all H atoms were calculated geometrically.

### Crystal data

**Table:**

[Al(CH_3_)_2_(C_17_H_30_N_3_)]	*D*_x_ = 1.095 Mg m^−^^3^
*M**_r_* = 333.49	Mo *K*α radiation, λ = 0.71073 Å
Orthorhombic, *P**b**c**n*	Cell parameters from 4606 reflections
*a* = 18.263 (4) Å	θ = 3.0–27.0°
*b* = 10.596 (2) Å	µ = 0.11 mm^−^^1^
*c* = 10.449 (2) Å	*T* = 293 K
*V* = 2022.0 (7) Å^3^	Block, colorless
*Z* = 4	0.40 × 0.30 × 0.30 mm
*F*(000) = 736	

### Data collection

**Table:**

Bruker SMART CCD area-detector diffractometer	1772 independent reflections
Radiation source: fine-focus sealed tube	1630 reflections with *I* > 2σ(*I*)
graphite	*R*_int_ = 0.059
phi and ω scans	θ_max_ = 25.0°, θ_min_ = 2.2°
Absorption correction: multi-scan (*SADABS*; Sheldrick, 1996)	*h* = −21→21
*T*_min_ = 0.959, *T*_max_ = 0.969	*k* = −12→7
7156 measured reflections	*l* = −11→12

### Refinement

**Table:**

Refinement on *F*^2^	Primary atom site location: structure-invariant direct methods
Least-squares matrix: full	Secondary atom site location: difference Fourier map
*R*[*F*^2^ > 2σ(*F*^2^)] = 0.095	Hydrogen site location: inferred from neighbouring sites
*wR*(*F*^2^) = 0.232	H-atom parameters constrained
*S* = 1.42	*w* = 1/[σ^2^(*F*_o_^2^) + (0.*P*)^2^ + 5.155*P*] where *P* = (*F*_o_^2^ + 2*F*_c_^2^)/3
1772 reflections	(Δ/σ)_max_ = 0.005
107 parameters	Δρ_max_ = 0.31 e Å^−^^3^
0 restraints	Δρ_min_ = −0.43 e Å^−^^3^

### Special details

**Table:**

Geometry. All esds (except the esd in the dihedral angle between two l.s. planes) are estimated using the full covariance matrix. The cell esds are taken into account individually in the estimation of esds in distances, angles and torsion angles; correlations between esds in cell parameters are only used when they are defined by crystal symmetry. An approximate (isotropic) treatment of cell esds is used for estimating esds involving l.s. planes.
Refinement. Refinement of F^2^ against ALL reflections. The weighted R-factor wR and goodness of fit S are based on F^2^, conventional R-factors R are based on F, with F set to zero for negative F^2^. The threshold expression of F^2^ > 2sigma(F^2^) is used only for calculating R-factors(gt) etc. and is not relevant to the choice of reflections for refinement. R-factors based on F^2^ are statistically about twice as large as those based on F, and R- factors based on ALL data will be even larger.

### Fractional atomic coordinates and isotropic or equivalent isotropic displacement parameters (Å^2^)

**Table:**

	*x*	*y*	*z*	*U*_iso_*/*U*_eq_	
Al	0.5000	0.29276 (18)	0.2500	0.0381 (6)	
N1	0.45266 (19)	0.1440 (3)	0.1850 (3)	0.0310 (8)	
N3	0.5000	−0.0569 (5)	0.2500	0.0397 (13)	
C1	0.5000	0.0706 (6)	0.2500	0.0318 (13)	
C2	0.3760 (2)	0.1118 (4)	0.1598 (4)	0.0334 (10)	
H2	0.3744	0.0415	0.0989	0.040*	
C3	0.3350 (3)	0.0733 (5)	0.2815 (5)	0.0418 (12)	
H3A	0.3575	−0.0019	0.3170	0.050*	
H3B	0.3392	0.1402	0.3444	0.050*	
C4	0.2546 (3)	0.0474 (5)	0.2557 (7)	0.0611 (15)	
H4A	0.2302	0.0270	0.3355	0.073*	
H4B	0.2501	−0.0247	0.1991	0.073*	
C5	0.2177 (3)	0.1614 (6)	0.1948 (6)	0.0569 (15)	
H5A	0.1673	0.1407	0.1743	0.068*	
H5B	0.2175	0.2309	0.2551	0.068*	
C6	0.2573 (3)	0.2010 (6)	0.0742 (5)	0.0539 (14)	
H6A	0.2349	0.2770	0.0405	0.065*	
H6B	0.2525	0.1352	0.0102	0.065*	
C7	0.3384 (2)	0.2255 (5)	0.1001 (5)	0.0435 (12)	
H7A	0.3626	0.2465	0.0203	0.052*	
H7B	0.3432	0.2972	0.1571	0.052*	
C14	0.4666 (3)	−0.1349 (4)	0.1484 (5)	0.0473 (13)	
H14A	0.4815	−0.1066	0.0641	0.057*	
H14B	0.4136	−0.1338	0.1538	0.057*	
C15	0.4971 (3)	−0.2657 (5)	0.1782 (6)	0.0613 (16)	
H15A	0.4642	−0.3314	0.1486	0.074*	
H15B	0.5447	−0.2777	0.1388	0.074*	
C18	0.4388 (3)	0.3932 (5)	0.3656 (6)	0.0586 (16)	
H18A	0.4076	0.3382	0.4141	0.088*	
H18B	0.4093	0.4505	0.3165	0.088*	
H18C	0.4695	0.4403	0.4229	0.088*	

### Atomic displacement parameters (Å^2^)

**Table:**

	*U*^11^	*U*^22^	*U*^33^	*U*^12^	*U*^13^	*U*^23^
Al	0.0304 (10)	0.0312 (10)	0.0526 (12)	0.000	−0.0093 (9)	0.000
N1	0.0254 (17)	0.0324 (18)	0.0351 (19)	−0.0015 (15)	0.0006 (15)	0.0007 (16)
N3	0.037 (3)	0.033 (3)	0.049 (3)	0.000	0.007 (3)	0.000
C1	0.032 (3)	0.032 (3)	0.032 (3)	0.000	0.010 (3)	0.000
C2	0.029 (2)	0.037 (2)	0.034 (2)	−0.0060 (18)	0.0002 (18)	−0.0059 (19)
C3	0.036 (2)	0.043 (3)	0.047 (3)	0.004 (2)	0.006 (2)	0.009 (2)
C4	0.033 (2)	0.057 (3)	0.094 (4)	−0.005 (2)	0.011 (3)	0.020 (3)
C5	0.026 (2)	0.061 (3)	0.084 (4)	−0.002 (2)	−0.001 (3)	0.006 (3)
C6	0.037 (3)	0.070 (4)	0.055 (3)	0.001 (3)	−0.014 (2)	0.000 (3)
C7	0.032 (2)	0.056 (3)	0.042 (2)	0.000 (2)	−0.006 (2)	0.013 (2)
C14	0.051 (3)	0.037 (3)	0.054 (3)	−0.009 (2)	0.018 (2)	−0.012 (2)
C15	0.057 (3)	0.036 (2)	0.091 (4)	−0.006 (3)	0.035 (3)	−0.012 (3)
C18	0.049 (3)	0.048 (3)	0.079 (4)	0.011 (3)	−0.019 (3)	−0.025 (3)

### Geometric parameters (Å, °)

**Table:**

Al—N1	1.922 (4)	C4—H4B	0.9700
Al—N1^i^	1.922 (4)	C5—C6	1.512 (8)
Al—C18^i^	1.961 (6)	C5—H5A	0.9700
Al—C18	1.961 (6)	C5—H5B	0.9700
N1—C1	1.346 (5)	C6—C7	1.528 (7)
N1—C2	1.465 (5)	C6—H6A	0.9700
N3—C1	1.351 (8)	C6—H6B	0.9700
N3—C14	1.477 (6)	C7—H7A	0.9700
N3—C14^i^	1.477 (6)	C7—H7B	0.9700
C1—N1^i^	1.346 (5)	C14—C15	1.526 (7)
C2—C7	1.520 (6)	C14—H14A	0.9700
C2—C3	1.531 (6)	C14—H14B	0.9700
C2—H2	0.9800	C15—C15^i^	1.505 (13)
C3—C4	1.517 (7)	C15—H15A	0.9700
C3—H3A	0.9700	C15—H15B	0.9700
C3—H3B	0.9700	C18—H18A	0.9600
C4—C5	1.522 (7)	C18—H18B	0.9600
C4—H4A	0.9700	C18—H18C	0.9600
			
N1—Al—N1^i^	69.8 (2)	C4—C5—H5A	109.5
N1—Al—C18^i^	119.0 (2)	C6—C5—H5B	109.5
N1^i^—Al—C18^i^	114.0 (2)	C4—C5—H5B	109.5
N1—Al—C18	114.0 (2)	H5A—C5—H5B	108.0
N1^i^—Al—C18	119.0 (2)	C5—C6—C7	111.3 (4)
C18^i^—Al—C18	114.2 (4)	C5—C6—H6A	109.4
C1—N1—C2	124.8 (3)	C7—C6—H6A	109.4
C1—N1—Al	90.4 (3)	C5—C6—H6B	109.4
C2—N1—Al	133.2 (3)	C7—C6—H6B	109.4
C1—N3—C14	124.0 (3)	H6A—C6—H6B	108.0
C1—N3—C14^i^	124.0 (3)	C2—C7—C6	112.1 (4)
C14—N3—C14^i^	112.0 (5)	C2—C7—H7A	109.2
N1^i^—C1—N1	109.4 (5)	C6—C7—H7A	109.2
N1^i^—C1—N3	125.3 (3)	C2—C7—H7B	109.2
N1—C1—N3	125.3 (3)	C6—C7—H7B	109.2
N1—C2—C7	108.7 (3)	H7A—C7—H7B	107.9
N1—C2—C3	112.3 (4)	N3—C14—C15	102.2 (5)
C7—C2—C3	109.3 (4)	N3—C14—H14A	111.3
N1—C2—H2	108.8	C15—C14—H14A	111.3
C7—C2—H2	108.8	N3—C14—H14B	111.3
C3—C2—H2	108.8	C15—C14—H14B	111.3
C4—C3—C2	111.9 (4)	H14A—C14—H14B	109.2
C4—C3—H3A	109.2	C15^i^—C15—C14	103.2 (3)
C2—C3—H3A	109.2	C15^i^—C15—H15A	111.1
C4—C3—H3B	109.2	C14—C15—H15A	111.1
C2—C3—H3B	109.2	C15^i^—C15—H15B	111.1
H3A—C3—H3B	107.9	C14—C15—H15B	111.1
C3—C4—C5	111.0 (4)	H15A—C15—H15B	109.1
C3—C4—H4A	109.4	Al—C18—H18A	109.5
C5—C4—H4A	109.4	Al—C18—H18B	109.5
C3—C4—H4B	109.4	H18A—C18—H18B	109.5
C5—C4—H4B	109.4	Al—C18—H18C	109.5
H4A—C4—H4B	108.0	H18A—C18—H18C	109.5
C6—C5—C4	110.9 (5)	H18B—C18—H18C	109.5
C6—C5—H5A	109.5		
			
N1^i^—Al—N1—C1	0.0	Al—N1—C2—C7	40.9 (5)
C18^i^—Al—N1—C1	106.9 (2)	C1—N1—C2—C3	51.3 (5)
C18—Al—N1—C1	−113.6 (2)	Al—N1—C2—C3	−80.2 (5)
N1^i^—Al—N1—C2	142.0 (5)	N1—C2—C3—C4	176.7 (4)
C18^i^—Al—N1—C2	−111.0 (4)	C7—C2—C3—C4	55.9 (5)
C18—Al—N1—C2	28.4 (5)	C2—C3—C4—C5	−56.6 (6)
C2—N1—C1—N1^i^	−146.9 (4)	C3—C4—C5—C6	55.6 (7)
Al—N1—C1—N1^i^	0.0	C4—C5—C6—C7	−55.1 (6)
C2—N1—C1—N3	33.1 (4)	N1—C2—C7—C6	−178.3 (4)
Al—N1—C1—N3	180.0	C3—C2—C7—C6	−55.4 (5)
C14—N3—C1—N1^i^	−158.0 (3)	C5—C6—C7—C2	56.0 (6)
C14^i^—N3—C1—N1^i^	22.0 (3)	C1—N3—C14—C15	167.3 (2)
C14—N3—C1—N1	22.0 (3)	C14^i^—N3—C14—C15	−12.7 (2)
C14^i^—N3—C1—N1	−158.0 (3)	N3—C14—C15—C15^i^	33.5 (6)
C1—N1—C2—C7	172.4 (4)		

Symmetry codes: (i) −*x*+1, *y*, −*z*+1/2.
